# Survival of women with breast cancer in Ottawa, Canada: variation with age, stage, histology, grade and treatment

**DOI:** 10.1038/sj.bjc.6601662

**Published:** 2004-02-24

**Authors:** A M Ugnat, L Xie, J Morriss, R Semenciw, Y Mao

**Affiliations:** 1 Surveillance and Risk Assessment Division, Centre for Chronic Disease Prevention and Control, Population and Public Health Branch, Health Canada, Ottawa, ON, Canada K1A 0K9; 2 Ottawa Regional Cancer Centre, Ottawa, ON, Canada K1H 8L6

**Keywords:** breast cancer, histology, prognosis, relative survival rate, TNM stage, treatment

## Abstract

This study examined the 5-year survival of 2192 breast cancer women diagnosed between 1994 and 1997 in Ottawa, Canada, by age, TNM stage, histology, grade and treatment, including assessment of the independent value of variables in defining prognosis. Our results showed that age, stage, treatment and grade significantly influenced outcome regardless of the confounding factors considered, with histology failing to achieve significant independent prognostic information. The survival rates were highest at ages 50–69 years for stage I and at ages 40–49 years for stages II–IV. The rates were lowest at ages ⩽39 years for stages I–II and at ages ⩾70 years for stages III–IV. The differences in survival between grade 1 and grade 3 were 9% in stage I and 20% in stage II. The treatment leading to the best survival was surgery plus radiation for stages I–II and surgery combined with chemotherapy for stages III–IV. Lobular carcinoma had a better prognosis than ductal carcinoma; this can be explained by more grade 1 and less grade 3 cases in lobular carcinoma. The worse prognosis for young patients than other ages can be explained by their higher proportion of poorly differentiated cancers. Stage I patients aged 50–69 years having the best survival is likely due to the earlier diagnosis achieved through screening.

Breast carcinoma is the most commonly diagnosed cancer (31% of total) in women in Canada. As a cause of cancer mortality in women, it is second only to lung carcinoma (25% lung *vs* 17% breast) (National Cancer Institute of Canada, 2002). Cancer survival is an essential component in cancer surveillance systems that support cancer prevention and control. Survival with breast cancer is associated with the value of prognostic factors such as stage, age, histology and grade, and the effectiveness of treatment. Numerous studies have documented the effect of age on 5-year survival with breast cancer, and debate continues concerning whether younger women have a poorer prognosis when diagnosed with breast cancer. A Five-year prognosis has been found to be poorer (Bonnier *et al*, 1995; Yildirim *et al*, 2000), better (La-Rosa *et al*, 1996) or the same (Barchielli and Balzi, 2000) for women diagnosed under age 35/40 years as compared with older women. Stage has been shown to be a strong predictor of outcome for female breast cancer, with survival declining as stage at diagnosis increases (Taylor and Coates, 1997; Zahl and Tretli, 1997). The fact remains, however, that wide variation in survival within stages does exist, depending on such additional factors as age, histology and grade. Histologic grade of a tumor can supply prognostic information in addition to that provided by LRD (localized, regional and distant) stage (Carriaga and Henson, 1995), but very few studies have examined the pattern of cancer survival as a function of grade within each TNM stage.

Multivariate analysis of data from a population-based cancer registry, namely Ottawa Region Cancer Centre (ORCC), is used here to examine the impacts of age and stage at diagnosis, histology, histologic grade and treatment on 5-year survival of female breast cancer in Ottawa, Canada.

## MATERIALS AND METHODS

Analysis was conducted on 2192 cases of primary breast cancer (International Statistical Classification of Diseases and Related Health Problems, 10th revision [ICD-10], code 174) registered by the ORCC between 1994 and 1997. The ORCC's population-based cancer registry covers all of Eastern Ontario and part of Western Quebec. The reporting system is based on pathology and cytology reports, clinical records and death certificates. This multiple reporting practice provides an accurate and complete set of data for each patient. Data regarding demographics, extent of disease, tumour histology and survival were available for the study period. All diagnoses were verified by histology. Patients were followed up to 18 April 2000, and their survival status was obtained by active follow-up, supplemented by passive follow-up involving record linkage to the Ontario Mortality Database. At the time of data analysis, we had survival information from 2181 patients (397 deaths, 1784 alive), and 11 (0.5%) were lost to follow-up. The median follow-up time was 45 months. Exclusion criteria were as follows: patients living in the province of Quebec at the time of diagnosis, diagnosis of cancer prior to lung cancer, report only by autopsy or death certificate and *in situ* carcinoma.

Covariates included in the study were age and TNM stage at diagnosis, histologic type, histologic grade and treatment. Tumour stage was coded using the Combined American Joint Committee on Cancer (AJCC) TNM classification system for disease stage at the time of diagnosis (American Joint Committee on Cancer, 1997). The AJCC system takes into account information on tumour size and tumour extension (T), regional lymph node involvement (N) and the presence of distant metastasis (M). The grouped TNM stage in this data included the pathologic stage group, augmented by the clinical stage group when the pathologic stage was not recorded. Age at cancer diagnosis was classified into four groups (0–39, 40–49, 50–69, 70 years and over). Treatment was classified as chemotherapy, radiotherapy, surgery, surgery with chemotherapy, surgery with radiation, chemotherapy plus radiotherapy (chemoradiation), surgery plus chemoradiation and no treatment (including both patients who were untreated and those with missing treatment informaion). The histology subtypes were grouped according to ICD-10 codes. Three histology subsets were defined: ductal carcinoma (8050, 8141, 8211, 8260, 8480, 8500, 8501, 8503, 8512 and 8530), lobular carcinoma (8520) and other miscellaneous histology types. The histologic grade is the degree of differentiation. Cases described as ‘well differentiated’ were assigned grade 1; ‘moderately differentiated,’ grade 2; ‘poorly differentiated,’ grade 3 and ‘ungraded (including grade Gx and missing data),’ grade unknown.

Patient characteristics were compared using the *χ*^2^ heterogeneity test for discrete data. All reported *P*-values were two-sided. Relative survival is the preferred method for analysing the survival of cancer patients in population studies. Relative survival rates (RSRs) adjust for competing causes of death that would be expected for persons of the same gender, age, period, and geographic region as the breast cancer patients in the study, without requiring information on the actual cause of death of each patient. Relative survival analysis was performed in RELSURV and Stata/Strel modules that both follow Estève's maximum likelihood method (Esteve *et al*, 1990). The expected survival rates were derived by single year of age up to 106 from the gender-specific Ontario life table for the study period. Statistical significance of each covariate was assessed by the likelihood ratio test, using the conventional level of 0.05. The goodness of fit for the models was evaluated on the basis of the deviances. The prognostic importance of TNM stage, histology, grade, age and treatment was analysed by both uni- and multivariate relative survival models. The multiple analysis was undertaken by taking into consideration all the prognostic factors examined in univariate analysis. First-order interaction terms were investigated for their effect on outcome by considering change in deviance or the likelihood ratio test. Relative excess risk (RER) was estimated from the relative survival model (Preston, 1998).

## RESULTS

The study showed that age, stage, grade and treatment had a significant effect on 5-year survival in both uni- and multivariate analyses, with histology failing to achieve significant independent prognostic information (Table 1

**Table 1:** Prognostic factor analysis of 2192 women with breast cancer, ORCC, 1994–1997

	**Univariate analysis**		**Multivariate analysis^a^**			**Univariate analysis**		**Multivariate analysis^a^**	
**Variables**	**RER (95% CI)**	***P*-value^b^**	**RER (95% CI)**	***P*-value^b^**	**Variables**	**RER (95% CI)**	***P*-value^b^**	**RER (95% CI)**	***P*-value^b^**
Stage		0.00000		0.00000	Age (years)		0.00005		0.00004
Stage I	1.00		1.00		0–39	1.00		1.00	
Stage II	4.54 (2.32,8.89)		3.05 (1.55,5.99)		40–49	0.45 (0.29,0.68)		0.46 (0.30,0.71)	
Stage III	15.64 (7.78,31.44)		8.28 (4.08,16.83)		50–69	0.29 (0.19,0.44)		0.45 (0.31,0.69)	
Stage IV	52.35 (26.74,82.47)		25.80 (12.88,51.65)		70+	0.47 (0.29,0.75)		0.85 (0.55,1.31)	
Treatment		0.00000		0.00592	Histology		0.00257		0.08866
Chemotherapy	1.00		1.00		Ductal carcinoma	1.00		1.00	
Radiotherapy	0.15 (0.06,0.40)		0.97 (0.42,2.23)		Lobular carcinoma	0.50 (0.24,1.01)		0.66 (0.37,1.16)	
Surgery	0.12 (0.06,0.25)		0.47 (0.24,0.94)		Others	1.76 (1.16,2.66)		1.18 (0.80,1.74)	
C+R^c^	0.74 (0.44,1.27)		1.07 (0.63,1.81)		Grade		0.00000		0.00007
S+C^c^	0.25 (0.18,0.36)		0.58 (0.39,0.86)		Grade 1	1.00		1.00	
S+R^c^	0.02 (0.01,0.12)		0.35 (0.15,0.81)		Grade 2	7.26 (1.77,29.72)		2.68 (1.00,7.15)	
S+C+R^c^	0.19 (0.11,0.30)		0.53 (0.33,0.85)		Grade 3	18.37 (4.55,74.16)		4.87 (1.84,12.87)	

Relative survival rate (RER) estimated according to regression models based on relative survival.

a Adjusted for all the other variables in the table.

b *P*-value from likelihood ratio test.

c C=chemotherapy; R=radiotherapy; S=surgery.

). Significant effect modifications between stage and age/treatment were also observed (*P*<0.001).

In Table 1, RERs and their 95% confidence intervals (CIs) are presented to assess the effect of each study variable on deaths due to breast cancer. A significant increase in relative risk was observed for stage IV diseases (relative risk of 52 compared to stage I in univariate analysis and 26 in multivariate study). Relative excess risk increases as grade goes from well-differentiated to poorly differentiated tumours. Women younger than 40 years have a relative risk over two times higher than women aged 50–69 years. The risk of dying was significantly lower for patients who received surgery or surgery plus radiotherapy than those who did not.

The overall 5-year RSR for 2192 women with breast cancer was 85%. The effect of each variable on survival in univariate analysis is shown in Table 2

**Table 2:** Relative 5-year survival of breast cancer patients by age group, TNM stage, histology, grade and treatment from univariate analysis, ORCC, 1994–1997

	**No. (%)^a^**	**Relative survival (%)**	
**Prognostic factor**	**observations**	**Rate**	**95% CI^b^**
*Age at diagnosis* *(years)*			
0–39	147 (6.7)	69.9	60.5–77.5
40–49	448 (20.4)	84.2	79.3–88.1
50–69	972 (44.3)	88.4	84.9–91.4
70+	625 (28.5)	81.3	74.9–86.2

*TNM stage*^c^			
Stage I	812 (37.0)	96.0	92.7–97.8
Stage II	843 (38.5)	85.6	81.7–88.7
Stage III	155 (7.1)	59.2	47.9–68.9
Stage IV	112 (5.1)	25.5	16.1–36.0

*Histology type*			
Ductal carcinoma	1765 (80.5)	84.32	81.6–86.7
Lobular carcinoma	233 (10.6)	91.02	81.0–95.9
Others	194 (8.9)	76.65	67.7–83.4

*Histologic grade*^c^			
G1 (well differentiated)	346 (15.8)	97.2	90.9–99.1
G2 (moderately differentiated)	674 (30.8)	87.8	83.7–91.0
G3 (poorly differentiated)	474 (21.6)	73.9	68.0–78.9

*Treatment*^c^			
Chemotherapy	38 (1.7)	46.2	25.2–64.8
Radiotherapy	54 (2.5)	87.7	73.1–94.7
Surgery	188 (8.6)	89.1	77.2–94.9
Chemotherapy+radiotherapy	135 (6.2)	61.4	51.3–70.1
Chemotherapy+surgery	396 (18.1)	82.1	75.8–86.9
Radiotherapy+surgery	389 (17.8)	96.0	89.7–98.6
Chemotherapy+radiotherapy+surgery	972 (44.3)	77.4	73.3–80.9

a *P*-value of *χ*^2^ test for the changes in a variable structure was less than 0.0001 for each variable considered.

b CI=confidence interval.

c Unstaged (%)=12.3, ungraded=31.8 and untreated or unknown=0.9.

. The 5-year survival declined significantly as stage at diagnosis increased. The 5-year RSRs were 96% for stage I, 86% for stage II, 59% for stage III and only 26% for stage IV. Patients younger than 40 years had the poorest 5-year prognosis, whereas patients diagnosed at ages 50–69 years experienced better survival than did women of other ages. Lobular carcinoma patients had a higher survival probability than ductal carcinoma patients through the follow-up. Significant survival declines were observed for grades of moderately differentiated (88%) and poorly differentiated (74%) compared to well differentiated (97%).

Within TNM stage, the presence of additional prognostic factors had a great impact on 5-year relative survival (Table 3

**Table 3:** Five-year relative survival for female breast cancer by TNM stage, age, histology, grade and treatment,^a^ ORCC, 1994–1997

	**TNM stage at diagnosis**							
	**I**		**II**		**III**		**IV**	
	**No**	**RSR (95% CI^b^)**	**No**	**RSR (95% CI)**	**No**	**RSRSR (95% CI)**	**No**	**RSR (95% CI)**
Age (years)								
0–39	34	91.1 (74.6,97.1)	81	68.6 (55.1,78.8)	13	48.7 (18.5,73.6)	8	28.6 (6.0,57.4)
40–49	137	92.8 (83.8,96.9)	187	90.0 (82.3,94.5)	45	71.2 (52.5,83.6)	25	33.2 (12.4,55.9)
50–69	410	97.9 (92.3,99.5)	351	88.6 (82.3,92.7)	56	60.2 (40.6,75.1)	38	27.9 (12.8,45.4)
70+	231	94.1 (79.7,98.4)	224	83.2 (71.9,90.2)	41	44.1 (22.3,64.0)	41	17.8 (6.6,33.2)

Histologic type								
Ductal carcinoma	687	95.8 (92.0,97.8)	679	85.1 (80.9,88.4)	112	51.4 (38.7,62.7)	69	31.1 (17.8,45.3)
Lobular carcinoma	81	99.9	85	90.1 (71.8,96.8)	28	89.9 (45.9,98.5)	13	—

Histologic grade								
G1 (well differentiated)	206	98.6 (91.2,99.8)	118	97.4 (75.5,99.8)	11	—	6	—
G2 (mostly differentiated)	296	94.6 (87.8,97.7)	295	87.4 (80.2,92.1)	48	69.7 (48.3,83.6)	17	—
G3 (poorly differentiated)	124	90.1 (75.6,96.2)	262	77.6 (69.6,83.7)	43	54.3 (35.1,70.1)	34	20.3 (4.5,43.9)

Treatment								
Chemotherapy	2	—	12	62.2 (25.7,84.7)	4	—	12	—
Radiotherapy	36	93.7 (70.9,98.8)	8	73.1 (25.5,93.1)	2	—	2	—
Surgery	100	90.7 (75.1,96.7)	46	85.9 (55.2,96.2)	2	—	2	—
C+R^c^	18	93.2 (46.8,99.3)	46	75.0 (57.6,86.1)	28	57.6 (32.8,76.2)	29	9.0 (1.5,25.0)
C+S^c^	89	82.3 (68.6,90.5)	204	86.2 (77.3,91.8)	14	69.9 (24.6,91.2)	26	27.9 (9.6,49.8)
R+S^c^	314	97.1 (89.8,99.2)	40	93.4 (49.9,99.3)	3	—	2	—
C+R+S^c^	247	92.8 (85.9,96.4)	482	80.6 (75.0,85.0)	101	53.0 (39.3,64.9)	36	21.9 (8.7,38.9)

a —=rate not reported. No. of cases less than 20 except for age; RSR=relative survival rate.

b CI=confidence interval.

c C=chemotherapy; R=radiotherapy; S=surgery.

). Age had a significant impact on survival within stage (*P*=0.0009). For earlier stage at diagnosis (I or II), women under 40 years had a much poorer RSR as compared with the other age groups, and patients diagnosed at ages 40–69 years experienced the best survival (50–59 years in stage I, and 40–49 years in stage II). For later stage (III or IV), patients aged 70 years and over had the poorest 5-year prognosis, whereas patients diagnosed at ages 40–49 years experienced better survival than did women of other ages. The observed survival advantage in lobular carcinoma patients was also seen within stage I, II and III. This would argue that the advantages seen were not attributable to possible stage distribution differences within histology groups. In addition, the discrepancies in survival between ductal and lobular carcinomas were small for stage I or II and much larger for stage III. Grading allowed the identification of high- and low-risk subgroups within each stage group. For each stage, survival rates decreased with advancing grade. The differences in survival between grade 1 and grade 3 were 9% for stage I and 20% for stage II. Therefore, poorly differentiated tumours, even at an early stage, could have an impact on survival.

The interaction of grade with age was also investigated (Table 4

**Table 4:** Five-year relative survival for female breast cancer by age at diagnosis and histologic grade, ORCC, 1994–1997

	**Histologic grade**					
	**G1 (well differentiated)**		**G2 (mostly differentiated)**		**G3 (poorly differentiated)**	
	**No**	**RSR (95% CI^a^)**	**No**	**RSR (95% CI^a^)**	**No**	**RSR (95% CI^a^)**
Age (years)						
0–39	11	82.5 (46.0,95.3)	43	81.2 (65.7,90.2)	55	57.2 (39.4,71.5)
40–49	49	No deaths	144	85.8 (76.7,91.5)	130	80.9 (70.1,88.1)
50–69	181	96.5 (85.5,99.2)	294	90.1 (83.8,94.0)	196	79.9 (70.7,86.5)
70 and older	105	96.9 (44.4,99.8)	193	84.5 (72.4,91.6)	93	58.8 (41.9,72.3)

a CI=confidence interval.

). Patients younger than 40 years had the poorest 5-year prognosis in each grade group. The RSRs were highest at ages 40–49 years in grade 1 group, and at ages 50–69 years in grade 2. For grade 3 disease, the RSRs were very close for women aged 40–49 years and for those aged 50–69 years as well as for those in the 0–39 and 70+ years age groups, but women in the two middle-age groups had substantially higher RSRs.

Treatment correlated significantly with patients’ survival (Table 2), and the interaction of treatment and stage was more predictive of outcome than any one factor separately (*P*=0.0003, Table 3). Patients with stage I or II breast cancer who were treated by surgery plus radiation were more likely to survival than those who received other treatments; patients with stage III or IV disease who received surgery plus chemotherapy had better survival.

## DISCUSSION

The TNM stage proved to be the most significant independent prognostic factor for determining survival. In our study series, around 75% of all patients were diagnosed in stages I and II. Regular mammography combined with regular clinical breast examination may offer the best opportunity to increase the percentage of early stage cases detected. The frequency distribution of stage and the 5-year relative survival rates by stage were similar to the data reported by the American Cancer Society (American Cancer Society, 2001). The observed decline in overall 5-year survival by stage was also seen within each histology or grade group. In all recent multiple regression analyses, stage had a major prognostic role. This is probably due to the fact that stage reflects the interaction between host and tumour. From this point of view, some variables connected with the host such as age, or connected with the tumour, such as histology and grade, could stand out as prognostic factors.

Histology was found to be prognostic in breast cancer. Lobular carcinoma had a better prognosis than ductal carcinoma for separate or combined stages in our study, while Mclaughlin *et al* (1995) had a similar result for combined stages. Although they could not account for the stage distribution differences within histology groups, these differences of survival can be explained as follows: grade 1 lesion was more common in women who had lobular carcinoma than in those who had ductal carcinoma (51 *vs* 20% among the graded cases); and conversely, grade 3 lesion was identified less frequently in lobular carcinoma (12 *vs* 34%).

Histologic grade was an important determinant of prognosis that also allowed risk stratification within a given tumour stage. The proportion of high-grade cases increased with advancing stage, whereas the percentage of low-grade cases decreased (Table 3). This distribution would suggest that tumour with advanced stage would be more likely to present at a higher grade. The survival rates decreased with advancing grade within each stage. This survival trend confirmed results reported by Carriaga and Henson (1995). For most breast cancers, histologic grade was a more meaningful prognostic feature than histologic classification, and helped to explain the favourable prognosis of most special histologic types. About 10% of low-grade tumours recurred within 5 years, compared with about 30% of high-grade cancers (International Union Against Cancer, 2001).

We found that overall survival was poorer for those younger than 40 years, and this difference was independent of tumour stage and type of treatment. This finding was consistent with other studies; for example Albain *et al* (1994) showed significantly worse prognosis for younger patients even after other prognostic factors were considered by multivariate analysis. The association of young age at diagnosis with a worse prognosis in our series can be explained by a higher proportion of poorly differentiated cancers (50%) as compared with other age groups (aged 40–49, 40; 50–69, 29; and 70+ years, 24%), suggesting an aggressive breast cancer phenotype. There are various possible explanations for the poor survival experience of young women with breast cancer: (1) lack of competing causes of death (Ederer *et al*, 1963); (2) higher frequency of undifferentiated tumours, more poorly differentiated cancer, microscopic lymph node involvement and negative hormonal receptor status (Bonnier *et al*, 1995) or (3) more cases diagnosed with stage II or III cancer (Gajdos *et al*, 2000). (In our series, 55% of tumours for women aged 0–39 years were diagnosed in stage II). Each of these hypotheses requires thorough investigation.

Our finding that patients aged 50–69 years had the best outcome can probably be explained by the relatively good local control (through mammography screening) for this age group in Canada, even though there is no significant difference in survival among age groups 40–49, 50–59 and 60–69 years (whose 5-year RSRs were 84, 88 and 88%, respectively). Some studies (Shapiro *et al*, 1982; Miller *et al*, 1992) have indicated that mammography screening has reduced breast cancer mortality and improved survival by detecting cancers at earlier, more treatable stages. In our study, nearly 96% of women with stage I cancers survived at least 5 years; this stage accounted for 50% of the women aged 50–69 years. In keeping with our results, Golledge *et al* (2000) described that patients aged 60–69 years experienced the best survival, although this favourable outcome was principally restricted to axillary lymph node negative patients.

Determining the effect of patient age on breast cancer prognosis is confounded by many factors, such as screening rates, menopausal status and differences in treatment. Consequently, significant differences in study designs have resulted in a lack of consensus regarding the prognostic effect of patient age. La-Rosa *et al* (1996) observed that women less than 35 years had a better prognosis at 5 years from diagnosis. Holli and Isola (1997) found that the 5-year RSR was highest in women aged 46–50 years, whereas they found no significant difference between younger and older age groups. A different finding for 5-year age-specific survival by stage was reported from the Rhode Island Tumor Registry, where women aged 40 years or less had a worse prognosis than other age groups, except for those with stage I disease (Chung *et al*, 1996). A limited number of young patients included in some studies, differences in patient selection, age grouping and analysis of outcome may contribute to the conflicting results for the relationship between age and prognosis.

In our study series, proportionally more stage I patients (39%) experienced surgery with radiotherapy, whereas more patients with stages II, III and IV diseases underwent surgery with radiotherapy and chemotherapy (57, 65 and 32%, respectively). Selection of therapy depends not only on the stage of the disease, but also on age, menopausal status, grade, histology, estrogen-receptor (ER) and progesterone-receptor (PR) status, HER2/neu gene amplification and general health. These factors may have resulted in selection bias in this study. Preventive mastectomy is an option to prevent breast cancer for women who are at very high risk for breast cancer. Possible candidates for this procedure are women with a strong family history of breast cancer and those who have a mutation in genes p53, BRCA1, or have gene BRCA 2. Chemotherapy can be considered for patients with hormone receptor-negative disease or advanced cancer. Hormonal therapy with or without chemotherapy is usually assigned for receptor-positive cancers.

Surgery plus radiotherapy plus chemotherapy would in general be given to higher risk patients (with four or more positive axillary nodes, large primary tumours, ER or PR negative, grade 2–3, 35 years of age and younger, etc.) than would surgery plus radiotherapy alone; this was confirmed by our study in terms of grade and age (the other variables were not included in this study) within each stage (Table 5

**Table 5:** Distribution of treatment by age at diagnosis, grade, histologic type and stage, ORCC, 1994–1997

	**Treatment**								
**Frequency (percentage)**	**Chemotherapy**	**Radiation**	**Surgery**	**C+R^a^**	**C+S^a^**	**R+S^a^**	**C+R+S^a^**	**No treatment**	***P*-value^b^**
Stage I (age, years)									0.01
0–39	0 (0.0)	2 (5.9)	3 (8.8)	2 (5.9)	5 (14.7)	8 (23.5)	14 (41.2)	0 (0.0)	
40–49	1 (0.7)	3 (2.2)	13 (9.5)	6 (4.4)	14 (10.2)	41 (29.9)	58 (42.3)	1 (0.7)	
50–69	1 (0.2)	22 (5.4)	50 (12.2)	5 (1.2)	37 (9.0)	168 (41.0)	125 (30.5)	2 (0.5)	
70 and older	0 (0.0)	9 (3.9)	34 (14.7)	5 (2.2)	33 (14.3)	97 (42.0)	50 (21.7)	3 (1.3)	
Stage II									<0.0001
0–39	1 (1.2)	0 (0.0)	2 (2.5)	7 (8.6)	10 (12.4)	1 (1.2)	60 (74.1)	0 (0.0)	
40–49	1 (0.5)	1 (0.5)	7 (3.7)	9 (4.8)	42 (22.5)	7 (3.7)	119 (63.6)	1 (0.5)	
50–69	2 (0.6)	5 (1.4)	12 (3.4)	17 (4.8)	87 (24.8)	11 (3.1)	217 (61.8)	0 (0.0)	
70 and older	8 (3.6)	2 (0.9)	25 (11.2)	13 (5.8)	65 (29.0)	21 (9.4)	86 (38.4)	4 (1.8)	
Stage III									0.002
0–39	0 (0.0)	1 (7.7)	0 (0.0)	3 (23.1)	0 (0.0)	0 (0.0)	9 (69.2)	0 (0.0)	
40–49	0 (0.0)	0 (0.0)	0 (0.0)	5 (11.1)	2 (4.4)	0 (0.0)	38 (84.4)	0 (0.0)	
50–69	1 (1.8)	1 (1.8)	1 (1.8)	14 (25.0)	2 (3.6)	0 (0.0)	36 (64.3)	1 (1.8)	
70 and older	3 (7.3)	0 (0.0)	1 (2.4)	6 (14.6)	10 (24.4)	3 (7.3)	18 (43.9)	0 (0.0)	
Stage IV									0.48
0–39	0 (0.0)	0 (0.0)	0 (0.0)	1 (12.5)	1 (12.5)	0 (0.0)	6 (75.0)	0 (0.0)	
40–49	3 (12.0)	0 (0.0)	1 (4.0)	10 (40.0)	7 (28.0)	0 (0.0)	4 (16.0)	0 (0.0)	
50–69	4 (10.5)	1 (2.6)	0 (0.0)	8 (21.1)	9 (23.7)	1 (2.6)	15 (39.5)	0 (0.0)	
70 and older	5 (12.2)	1 (2.4)	1 (2.4)	10 (24.4)	9 (22.0)	1 (2.4)	11 (26.8)	3 (7.3)	

Stage I (grades)									<0.0001
G1	0 (0.0)	9 (4.4)	35 (17.0)	2 (1.0)	13 (6.3)	108 (52.4)	38 (18.5)	1 (0.5)	
G2	1 (0.3)	17 (5.7)	29 (9.8)	5 (1.7)	34 (11.5)	110 (37.2)	98 (33.1)	2 (0.7)	
G3	0 (0.0)	1 (0.8)	11 (8.9)	7 (5.7)	19 (15.3)	26 (21.0)	59 (47.6)	1 (0.8)	
Stage II									0.04
G1	2 (1.7)	1 (0.9)	9 (7.6)	5 (4.2)	34 (28.8)	7 (5.9)	58 (49.2)	2 (1.7)	
G2	1 (0.3)	1 (0.3)	16 (5.4)	18 (6.1)	65 (22.0)	14 (4.8)	179 (60.7)	1 (0.3)	
G3	1 (0.4)	2 (0.8)	14 (5.3)	15 (5.7)	61 (23.3)	10 (3.8)	158 (60.3)	1 (0.4)	
Stage III									0.19
G1	0 (0.0)	0 (0.0)	0 (0.0)	4 (36.4)	1 (9.1)	0 (0.0)	6 (54.6)	0 (0.0)	
G2	1 (2.1)	0 (0.0)	1 (2.1)	5 (10.4)	8 (16.7)	1 (2.1)	32 (66.7)	0 (0.0)	
G3	0 (0.0)	0 (0.0)	1 (2.3)	5 (11.6)	2 (4.7)	2 (4.7)	33 (76.7)	0 (0.0)	
Stage IV									0.06
G1	1 (16.7)	0 (0.0)	0 (0.0)	0 (0.0)	3 (50.0)	0 (0.0)	2 (33.3)	0 (0.0)	
G2	1 (5.9)	0 (0.0)	1 (5.9)	2 (11.8)	4 (23.5)	1 (5.9)	8 (47.1)	0 (0.0)	
G3	1 (2.9)	0 (0.0)	1 (2.9)	6 (17.7)	11 (32.4)	0 (0.0)	15 (44.1)	0 (0.0)	

Stage I (histologic type)									0.51
Ductal carcinoma	1 (0.2)	30 (4.4)	82 (11.9)	16 (2.3)	73 (10.6)	269 (39.2)	212 (30.9)	4 (0.6)	
Lobular carcinoma	1 (1.2)	4 (4.9)	14 (17.3)	2 (2.5)	10 (12.4)	25 (30.9)	23 (28.4)	2 (2.5)	
Stage II									0.52
Ductal carcinoma	10 (1.5)	7 (1.0)	31 (4.6)	37 (5.5)	165 (24.3)	30 (4.4)	394 (58.0)	5 (0.7)	
Lobular carcinoma	1 (1.2)	0 (0.0)	7 (8.2)	2 (2.4)	23 (27.1)	6 (7.1)	46 (54.1)	0 (0.0)	
Stage III									0.9
Ductal carcinoma	4 (3.6)	2 (1.8)	2 (1.8)	20 (17.9)	11 (9.8)	2 (1.8)	70 (62.5)	1 (0.9)	
Lobular carcinoma	0 (0.0)	0 (0.0)	0 (0.0)	4 (14.3)	2 (7.1)	0 (0.0)	22 (78.6)	0 (0.0)	
Stage IV									0.02
Ductal carcinoma	8 (11.6)	0 (0.0)	0 (0.0)	15 (21.7)	19 (27.5)	1 (1.5)	26 (37.7)	0 (0.0)	
Lobular carcinoma	1 (7.7)	0 (0.0)	0 (0.0)	5 (38.5)	4 (30.8)	0 (0.0)	3 (23.1)	0 (0.0)	

a C=chemotherapy; R=radiotherapy; S=surgery.

b *P*-value of *χ*^2^ test.

). In addition, radiotherapy might have been given post mastectomy to patients with higher risk profiles within a given stage, or with breast-conserving surgery to lower risk patients. Treatment protocols were significantly linked to the age at diagnosis for stage I, II and III (*P*⩽0.01, Table 5): the percentage of patients who received the three combined treatments mainly decreased as age increased. Treatment differences between younger and older patients may be partially attributed to the poor physical condition and the comorbid diseases of the older patients. Histology was not significantly related to selecting treatment for stage I, II and III (Table 5).

Hormonal positive status predicts response to hormonal therapy. Many studies have shown that women with ER- or PR-positive cancers have a better prognosis than patients whose cancers do not have these receptors. An interaction of treatment with receptor status may partially explain the inferior results for chemotherapy-treated patients. However, since hormonal receptor status was missing for 93% of the patients in this series, the impact of hormonal receptor status on outcome could not be accurately assessed.

There are some other limitations to the study. Firstly, in the assessments of multiplicative effects of patient characteristics (i.e. interactions), the study did not have adequate power to produce conclusive evidence for the study subgroups with small sample size or inadequate end points (numbers of deaths). However, the results were suggestive and can provide direction for future research. For example, in the calculation of RSRs by age group for patients with stage I cancer, the RSR estimates were not reliable because of either the low frequency of death in this group or the small number of patients in the youngest age subgroup, so they need to be re-evaluated in a large series. To compensate for such a deficit in expected sample size and end points, two major strategies were used in this study: first, collapsing two adjacent groups into one group and, second, reducing the number of intervals during the analysis. Secondly, since information on cause of death was missing for 86% of the individuals in this series, we used a relative survival model, but the results could not be confirmed by Cox proportional regression. However, the estimate of relative survival is closer in theory to net survival, and empirical evidence shows that relative survival approximates net survival more closely than other methods (Esteve *et al*, 1990).

An important strength of this study is the ability to investigate more comprehensively the impact of demographic, histologic and therapeutic factors on survival with breast cancer. The size of the study is sufficiently large to examine effect modifications and perform survival analysis across different subgroups of breast cancer. Perhaps more importantly, the results will provide further evidence for the debates on age influences on survival and the importance of grade on prognosis. In addition, one-quarter of patients diagnosed with overt metastases were alive at 5 years. The data thus provide fairly clear circumstantial evidence that the society, which puts resources and energy into breast cancer prevention and control through supporting integrated policy development, surveillance, research, education, diagnosis and treatment, has seen improved results. The development of new chemotherapeutic agents, the use of new radiation techniques and the implementation of multimodality therapy in advanced disease (81% of stage IV patients received chemotherapy combined with radiation and/or surgery in this study) have been observed to improve survival in patients with more advanced stages of disease.

In conclusion, our study has found that age and TNM stage at diagnosis, histologic grade and treatment were independent significant prognostic factors for breast cancer, whereas histologic subtype was statistically significant in the univariate analysis but not after adjusting simultaneously for other prognostic factors. Even more information was obtained when prognostic factors were examined in combination. Within stage, significantly wide variation in survival was seen due to age or treatment. The fact that lobular carcinoma had a better prognosis than ductal carcinoma can be explained by more grade 1 and less grade 3 cases in lobular carcinoma. The worse prognosis for young patients than other ages can be explained by their higher proportion of poorly differentiated cancers. Stage I patients aged 50–69 years having the best survival may be due to the earlier diagnosis achieved through screening. Although the analyses involved small sample of some categories, our findings support the existing literature concerning the prognostic effects of those factors. The determination of prognosis depends on the accurate assessment of prognostic factors and the appropriate choice of therapeutic and supportive intervention. Further studies are needed to confirm the results in a large sample and possibly to ensure the inclusion of other factors such as menopausal status, oestrogen receptor levels, progesterone receptor levels, number of nodes and waiting time for treatment.
